# The Negative Correlation between Fiber Color and Quality Traits Revealed by QTL Analysis

**DOI:** 10.1371/journal.pone.0129490

**Published:** 2015-06-29

**Authors:** Hongjie Feng, Lixue Guo, Gaskin Wang, Junling Sun, Zhaoe Pan, Shoupu He, Heqin Zhu, Jie Sun, Xiongming Du

**Affiliations:** 1 Institute of Cotton Research of Chinese Academy of Agricultural Sciences/State Key Laboratory of Cotton Biology, Anyang, China; 2 College of Agriculture, The key Laboratory of Oasis Eco-Agriculture, Xinjiang Production and Construction Group, Shihezi University, Shihezi, China; Zhejiang A & F university, CHINA

## Abstract

Naturally existing colored cotton was far from perfection due to having genetic factors for lower yield, poor fiber quality and monotonous color. These factors posed a challenge to colored cotton breeding and innovation. To identify novel quantitative trait loci (QTL) for fiber color along with understanding of correlation between fiber color and quality in colored cotton, a RIL and two F_2_ populations were generated from crosses among Zong128 (Brown fiber cotton) and two white fiber cotton lines which were then analyzed in four environments. Two stable and major QTLs (*q*LC-7-1, *q*FC-7-1) for fiber lint and fuzz color were detected accounting for 16.01%-59.85% of the phenotypic variation across multiple generations and environments. Meanwhile, some minor QTLs were also identified on chromosomes 5, 14, 21 and 24 providing low phenotypic variation (<5%) from only F2 populations, not from the RILs population. Especially, a multiple-effect locus for fiber color and quality has been detected between flanking markers NAU1043 and NAU3654 on chromosome 7 (A genome) over multiple environments. Of which, *q*LC-7-1, *q*FC-7-1 were responsible for positive effects and improved fiber color in offsprings. Meanwhile, the QTLs (*q*FL-7-1, *q*FU-7-1, *q*FF-7-1, *q*FE-7-1, and *q*FS-7-1) for fiber quality had negative effects and explained 2.19%-8.78% of the phenotypic variation. This multiple-effect locus for fiber color and quality may reveal the negative correlation between the two types of above traits, so paving the way towards cotton genetic improvement.

## Introduction

Naturally prevailing colored cotton is a “natural pigment fiber” that displays brown or green color [1] and is an environmental and health-friendly fiber because it does not require dying [2]. However, the existing natural colored cotton genotypes have lower yield, poor fiber quality and monotonous color, all of which pose a challenge for colored cotton breeding and innovation [3–5]. Previous breeding experiments showed that it was difficult to have stable and uniform fiber color while maintaining a high yield and good fiber quality. Marker-assisted selection (MAS) offers a new avenue for efficient genetic improvement of colored cotton.

Most studies on naturally colored cotton considered that fiber color is conditioned by a major gene with incomplete dominance in inheritance [6–7], while some other studies showed that the inheritance of fiber color is due to the effect of two or more genes [8]. In addition to this, there may be some minor genes that played an additional role in the inheritance [7]. Though many scientists had studied the inheritance of naturally occurring colored cotton, but mostly are on the aspect of fiber lint coloring only, while less reports have been presented regarding the combined genetic analyses with lint and fuzz as well. Shi *et al*. [3] concluded that colored cotton fiber lint and fuzz are controlled by one pair of major genes incomplete in dominance on non-homologous chromosomes, respectively. Green fiber lint and fuzz were respectively dominant to brown ones, while brown lint and fuzz were dominant to white ones, respectively. There exists genetic interaction between lint and fuzz coloring genes. Further, it was indicated that fiber color had significant negative correlation with lint percentage, fiber length, uniformity, elongation and strength through the complete diallel-cross analysis among two brown cotton cultivars and three white cotton cultivars [9]. As to color cotton, Ahuja *et al*. [10] reported that there were significant variations between parents and F_1_ in terms of agronomic traits and fiber quality. Meanwhile, the lower concentrations of carbohydrates and cellulose decreased yield and quality in colored cotton than that in white cotton [5].

Cotton fiber quality is quantitative trait controlled by multiple genes, which are vulnerable to environmental impact. An individual QTL is described as ‘major’ or ‘minor’ on the basis of the proportion of the phenotypic variation explained by a QTL (based on the R^2^ value). Sometimes, major QTL may refer to as major that is stable across multiple environments whereas minor QTL is preferably referred to as minor that is environmentally unstable [11]. For white fiber cotton, numerous major and minor QTLs for fiber quality traits were identified and assigned to chromosomes of sub-genomes using inter-specific populations from crosses between *G*. *hirsutum* and *G*. *barbadense* [12–15] and in intra-specific *G*. *hirsutum* populations [16–19]. In the colored cotton, thirteen QTLs for fiber quality traits were detected by performing multiple-interval mapping in a cross between brown cotton T586 and white cotton Yumian 1. Five out of thirteen QTLs (FL1 and FU1 on chromosome 6, FL2, FU2 and FF1 on chromosome 7, which include a maker Lc1 related to brown fiber color gene) were observed over five environments [19]. However, a few scholars pay attention to the QTL mapping of fiber color and quality in colored fiber cotton, simultaneously. In this study, we structured a recombinant inbred line (RILs) population which was derived from a cross between a brown and a white upland cotton to detect and characterize QTLs for fiber color and quality traits in different environments. A comparative analysis of the genetic maps and QTL tagging for fiber color and quality traits was then performed between the RILs and two F_2_ populations in which one of F_2_ populations was derived from the same cross as that of RILs. It will facilitate the understanding of correlation between fiber color and quality in colored cotton.

## Materials and Methods

### Plant materials

Three upland cotton lines were used to construct the mapping populations. The germplasm lines used included Zong128 of brown fibers with poor fiber quality, and two others KucheT94-4 and Liao96-23-30 of white fibers with high quality. A population of 245 recombinant inbred lines (RILs) and a population of 267 F_2_ individuals were developed by crossing Zong128 with KucheT94-4 while Zong128 and Liao96-23-30 were crossed to develop another F_2_ population of 247 individuals.

### Field design

The parental lines and 245 RILs were evaluated for fiber color and quality traits in Anyang station (36° 10′N, 114° 35′E) Henan province (Yellow River valley), in 2009 and 2010 (Env. 1 and Env. 2, respectively), in Huangmei station (30° 04′N, 115° 56′E) Hubei province (Yangtze River valley), in 2010 (Env. 3), and in Shihezi University (44° 18′N, 86° 00′E), Xinjiang province (Northwest region), China, in 2010 (Env. 4). The three ecological zones represent important commercial cotton production regions in China. The F_2_ populations were planted in Anyang station only for the assessment of fiber color and quality. Field tests were performed by arranging randomized complete block design with three replicates keeping each plot 4m long with row spacing of 0.75 m.

Seed cotton was harvested from all plants within each plot, and ginned on a laboratory gin. A fiber lint sample (15 g) from each plot was sent to Supervision Inspection & Testing Center of Cotton Quality, Ministry of Agriculture (Anyang, Henan), for measurements of fiber length, strength, micronaire, length uniformity and elongation by HVI 900 fiber testing system. Quantitative measurements of fiber lint and fuzz color were performed by the color coefficient calculated by the following equation P = R/(G+B) (R: red, G: green, B: brown), which the quantitative value of fiber color increases proportionally with the fiber darkness [9].

### Assay of DNA markers

Total DNA of the parents, each F_2_ individual and of RI lines was isolated from fresh leaf tissues by CTAB method [20]. A total of 5780 simple sequence repeats (SSRs) markers were used to detect polymorphisms, by following sources: BNL (Brookhaven National Laboratory, NY); JESPR [21]; TM and MGHES (USDA-ARS, Crops Germplasm Research Unit, Texas); CIR (French Agricultural Research Centre for International Development, FRA) [22]; CM; MUCS and MUSS (University of California Davis, USA); DPL (Delta and Pine Land, USA) and NAU (Nanjing Agricultural University, China) [23], GH, HAU, CSHES. The PCR reaction profile was initial denaturation of 94°C for 2 min, followed by 35 cycles of 30s at 94°C for denaturation, 30s at 52°C for annealing, 30s at 72°C for extension and 5 min at 72°C for final extension after the last cycle. The amplified PCR products were separated on 8% (w/v) denaturing polyacrylamide gel and visualized by silver staining [24]. The polymorphic SSR markers were integrated into the map constructed by Zhang et al. [25].

### Genetic map construction

Linkage analysis was conducted using JoinMap 4.0 [26] with a recombination frequency of 0.40 and a LOD score of 3.0 for the RIL population. A LOD threshold of 5.0 was used for two F_2_ populations due to severely skewed segregation ratios in F_2_ populations. The Kosambi mapping function was used to convert the recombination frequencies to map distances [27]. Linkage groups were localized to chromosomes using previously anchored SSR markers [14, 22, 28–34] and the source information of SSR primers was taken from DPL (http://www.cottonmarker.org/).

### QTL mapping

Phenotypic data were described (position, distribution) and compared using the statistical software SPSS 20 (Release 20.0.0, IBM, 2011). The trait averages of different populations were compared by General Linear Model The package Windows QTL Cartographer 2.5 was applied to identify QTLs by composite interval mapping (CIM) [35–37]. The parameters were set at 2.0 cM mapping step, 5 control markers, and 1,000 permutation tests. QTLs were declared significant if the corresponding likelihood ratio (LR) score was greater than 11.5 (equal to a LOD score of 2.5). The percent phenotypic variance (PV) explained by a QTL was estimated at the highest probability peaks. Graphic representation of the linkage groups and QTL was created by MapChart 2.2 [38].

The chromosome information of QTL nomenclature was consistent with the previous chromosome naming system [39]. QTLs for the fiber traits were named as follows: *q*+trait-Chromosome-No. [40] where traits were: lint color (LC), fuzz color (FC), fiber length (FL), fiber length uniformity (FU), fiber strength (FS), fiber elongation (FE) and fiber fineness (micronaire value, FF).

## Results

### Performances of fiber color and quality trait components of the RIL and the different F_2_ populations

The phenotypic data for fiber color and quality of the three populations and the parents were summarized in S1 Table. The brown fiber parent (Zong128) differed in LC, FC, FL, FU, FF, FE, and FS from the two white fiber parents (KucheT94-4 and Liao96-23-30). Broad variations for all the traits were observed among the three populations, and transgressive segregations existed in every population. Interestingly, the average color of fiber lint and fuzz in the RIL was similar to those in the F_2_ populations of Zong128 × KucheT94-4, but both the lint and fuzz color in the F_2_ populations of Zong128 × Liao96-23-30 were lighter than that in the RIL and F_2_ of Zong128 × KucheT94-4 (S1 Table). These results indicated that the genotype of the parent with the white fiber also determined the fiber color of the offspring. A distribution with a significant negative kurtosis for the LC and FC in the four environments in the RIL population indicated that the observation clusters had very short tails. However, these two traits in the two F_2_ populations of Zong128 × KucheT94-4 and Zong128 × Liao96-23-30 were a normal distribution. The other traits showed a normal distribution in all the environments and were suitable for QTL analysis. Although the LC and FC in the RIL population were not normally distributed, it could be analyzed by the QTL technique, which has been discussed in this paper.

**Fig 1:**
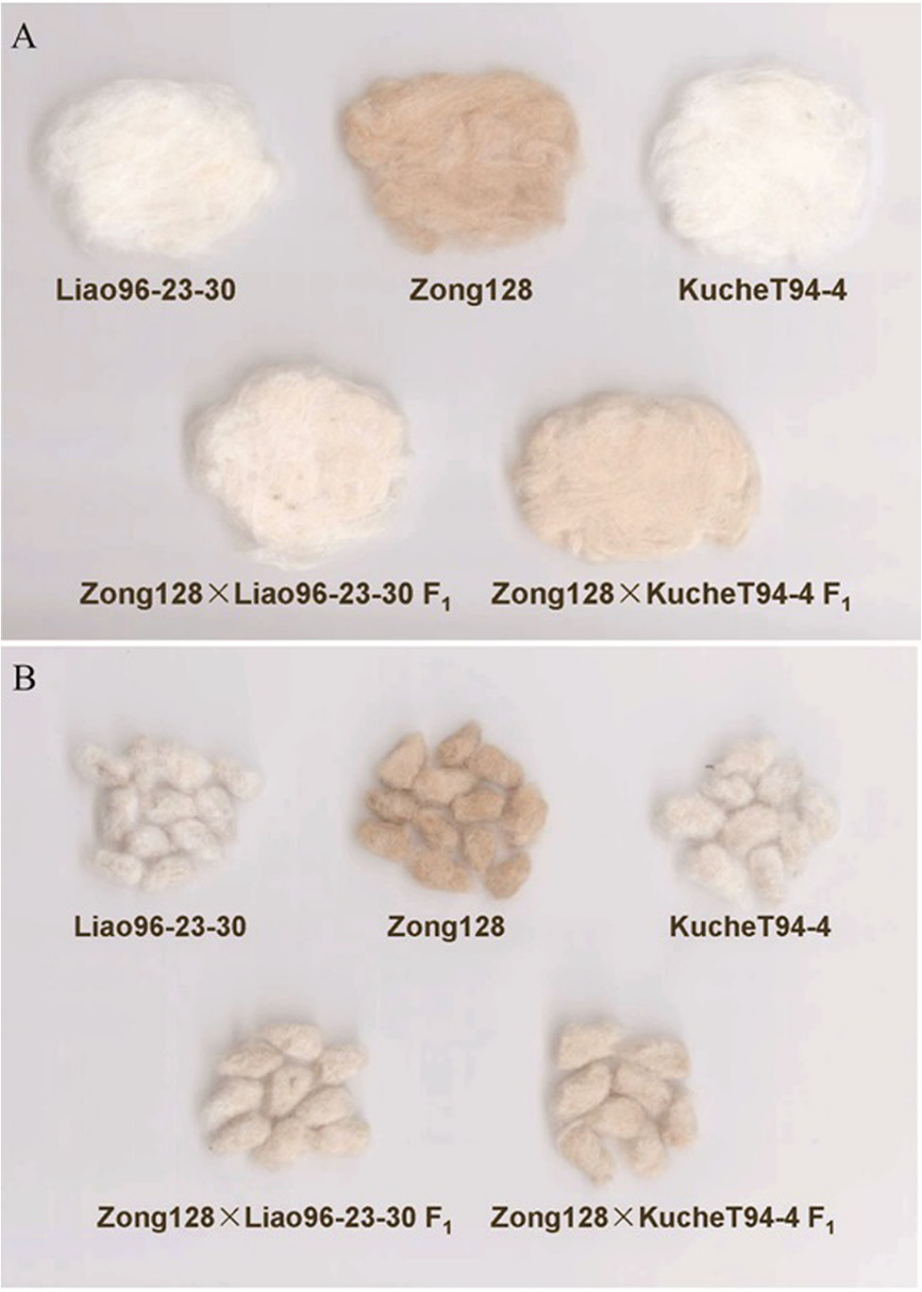
A. the lint colored of parents and F_1_ generation; B: fuzz color of parents and F_1_ generation.

The fiber color of F_1_ from the cross between Zong128 and KucheT94-4 is light brown (Fig 1), while the fiber color of F_1_ from the cross between Zong128 and Liao96-23-30 is nearly white (Fig 1).

### Construction of the linkage maps

**Fig 2:**
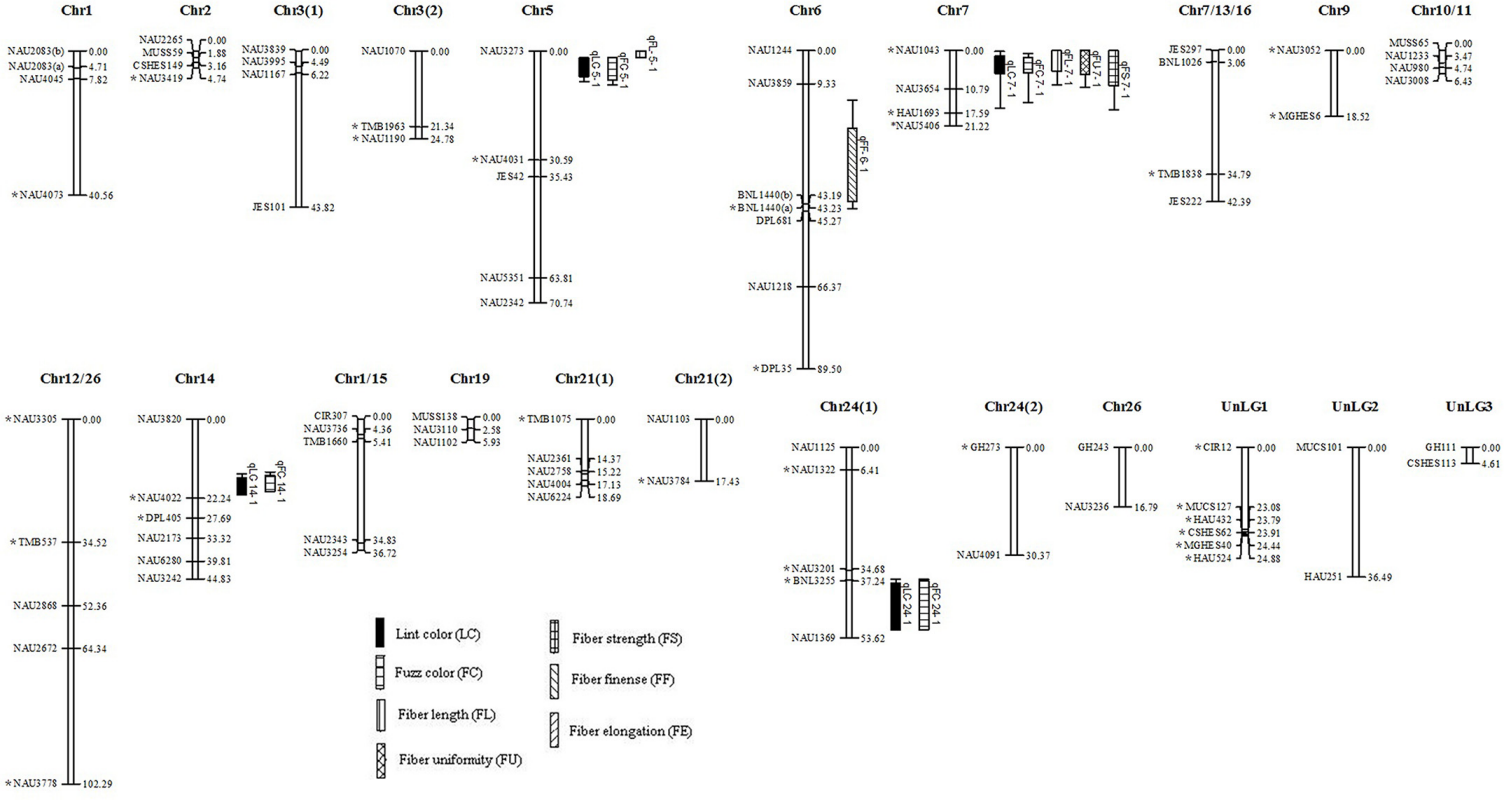
Linkage map from RIL population of Zong128 × KuchuT94-4. Linkage groups that could not be assigned to a chromosome were given the preliminary names UnLG1 –UnLG5. Bars and lines on the right-hand side of the linkage groups show the QTL likelihood intervals. Map distances in centimorgans (cM) are indicated on the left-hand side of the linkage groups. LC, lint color; FC, fuzz color; FL, fiber length; FU, fiber uniformity; FF, micronaire; FE, fiber elongation; FS, fiber strength; *Indicates distorted markers.

**Fig 3:**
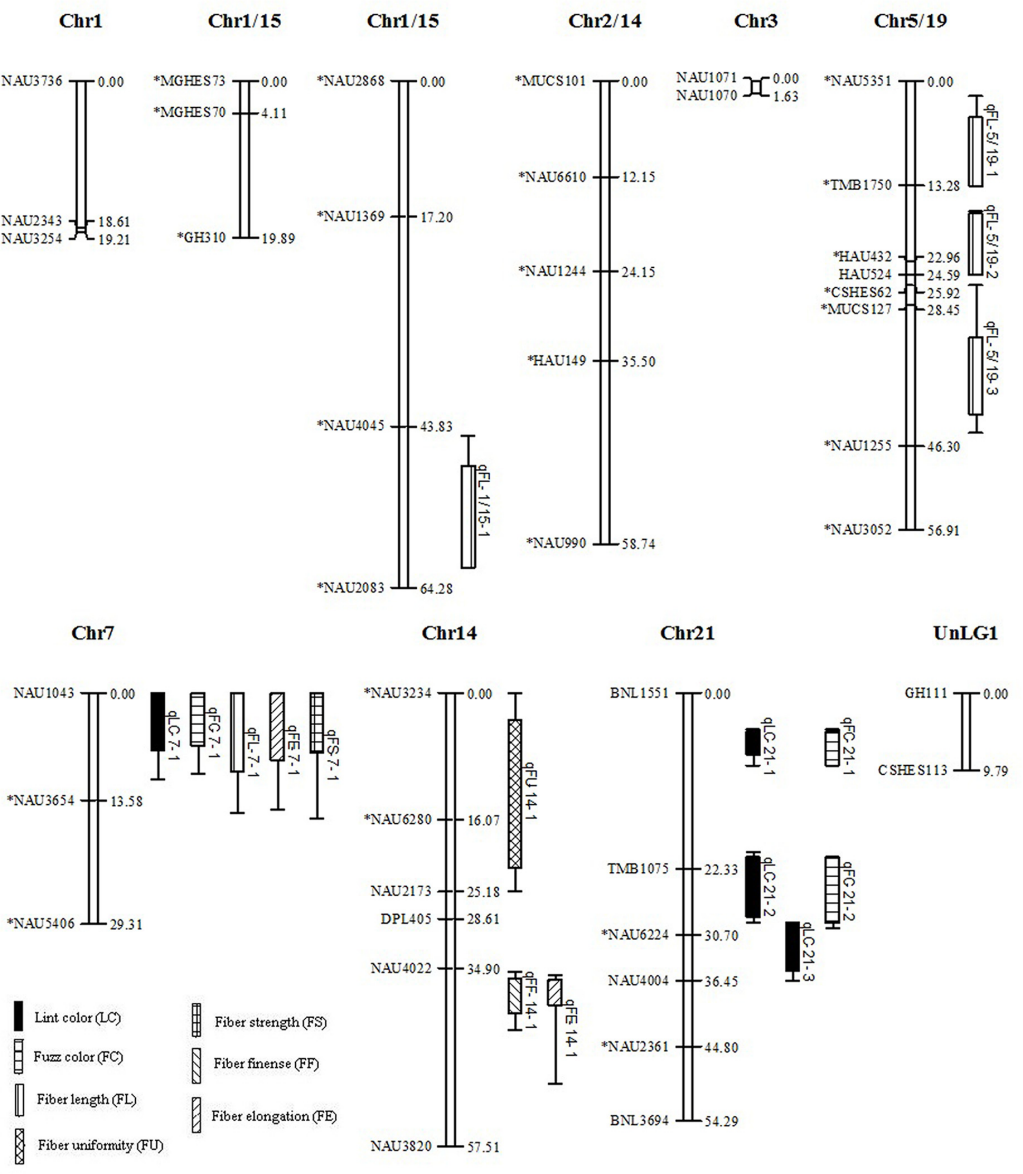
Linkage map from F_2_ population of Zong128 × KuchuT94-4. Linkage groups that could not be assigned to a chromosome were given the preliminary names UnLG1 UnLG2 and UnLG3. Bars and lines on the right-hand side of the linkage groups show the QTL likelihood intervals. Map distances in centimorgans (cM) are indicated on the left-hand side of the linkage groups. LC, lint color; FC, fuzz color; FL, fiber length; FU, fiber uniformity; FF, micronaire; FE, fiber elongation; FS, fiber strength; *Indicates distorted markers.

**Fig 4:**
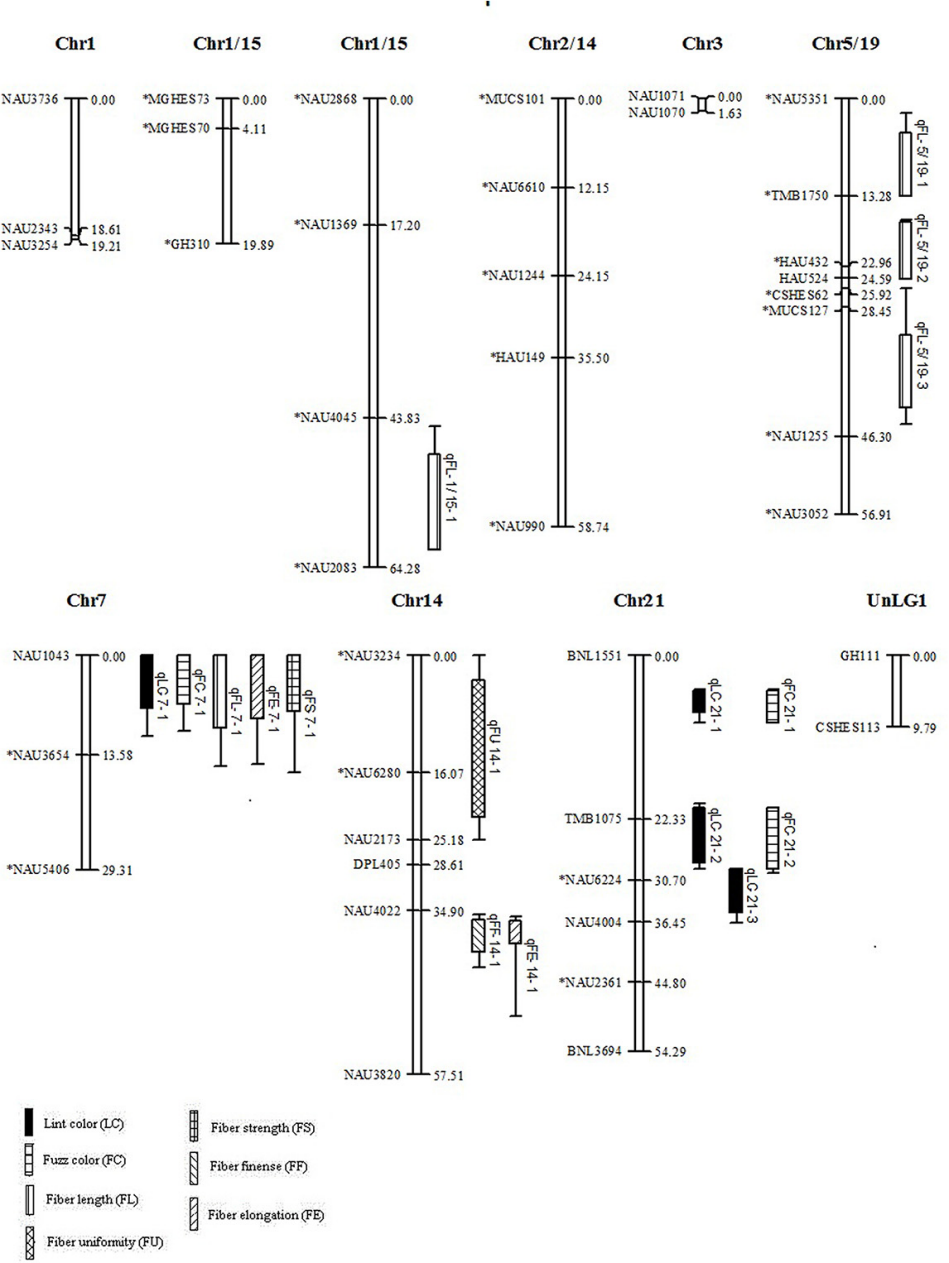
Linkage map from F_2_ of Zong128 × Liao96-23-30 population. Linkage groups that could not be assigned to a chromosome were given the preliminary names UnLG1. Bars and lines on the right-hand side of the linkage groups show the QTL likelihood intervals. Map distances in centimorgans (cM) are indicated on the left-hand side of the linkage groups. LC, lint color; FC, fuzz color; FL, fiber length; FU, fiber uniformity; FF, micronaire; FE, fiber elongation; FS, fiber strength; *Indicates distorted markers.

In total, 110 of the 5780 SSR primer pairs (1.9%) showed polymorphism between the Zong128 and KucheT94-4 at 116 loci across the genome. The number of polymorphic markers from BNL, NAU, TMB, CIR, GH, HAU, MGHES, MUCS, JESPR, DPL, CSHES and other sources was 5, 59, 6, 2, 12, 6, 2, 6, 4, 6, 3, 5 respectively in the RIL population. Of these, 89 loci were assigned to 27 linkage groups (Fig 2) with a total map distance of 481.54 cM covering approximately 10.82% of the total recombination length of the cotton genome [30]. A total of 86 loci were assigned to 22 linkage groups (Fig 3) with a total map distance of 755.35 cM in the F_2_ population of Zong128 × KuchuT94-4. The map covered approximately 16.97% of the total recombination length of the cotton genome. From the 70 primer pairs that amplified polymorphism in the F_2_ population of Zong128 × Liao96-23-30, 42 were assigned to 10 linkage groups (Fig 4) with a total map distance of 369.93 cM which covered approximately 8.30% of the total recombination length of the cotton genome. In this study, the number of markers and genome coverage was lower due to the extremely low genetic diversity within Upland cotton. But this did not influence the objective of this experiment since we mainly focused on understanding the fiber color variation, and were addressing the fundamental issues related to the contradictive association between fiber color and fiber quality in cotton.

Severe segregation distortion was observed in RILs (48 loci out of 116, 41.38%) and in the corresponding F_2_ population (50 loci out of 116, 43.10%) at a significance level P < 0.05. 22 loci (45.65%) and 26 loci (54.35%) deviated respectively toward Zong128 and KuchuT94-4 in the RILs population (Fig 2). Similarly, 19 loci (40.00%) had an excess of Zong128 alleles while 31 loci (62.00%) had an excess of KuchuT94-4 alleles in the F_2_ population (Fig 3). 36 loci out of 70 (51.43%) showed segregation distortion in the F_2_ population of Zong128 × Liao96-23-30, at a significance level P < 0.05. Of these, 11 loci out of 36 (30.56%) had an excess of Zong128 alleles and 25 loci (69.44%) had an excess of Liao96-23-30 alleles (Fig 4).

### QTL analysis for fiber color and quality in RIL population

#### Fiber color

Two major QTLs for fiber color were detected (Table 1, Fig 2) between NAU1043 and NAU3654 on chromosome 7 in four different environments. One lint color QTL (*q*LC-7-1) was 1.0 cM apart from the marker NAU1043 (co-dominant in parents and F_1_), which explained 42.83% (LOD 10.34), 49.82% (LOD 10.34), 43.16% (LOD 9.62), and 37.88% (LOD 9.63) of the phenotypic variation (PV) in the four environments, respectively. The alleles of this QTL were derived from the brown fiber parent Zong128, due to the positive additive effect of 3.075, 3.360, 2.976, and 2.78 in the four environments, respectively. Another QTL (*q*FC-7-1) related to fuzz color was located 2.01 cM apart from the marker NAU1043, which exhibited 50.19% (LOD 9.47), 59.85% (LOD 9.31), 49.91% (LOD 9.69), and 40.05% (LOD 8.58) PV in four environments, respectively. The fuzz color allele derived from the brown fiber parent Zong128, resulted in the positive additive effect of 3.235, 3.484, 3.540, and 2.915 in four environments, respectively. The results showed that the two QTLs were stable and major QTLs for the fiber color due to their constant and well PV across multiple environments.

**Table 1:** QTLs detected for fiber color and quality traits form RIL population of Zong128 × KuchuT94-4 in more than one environment with composite interval mapping.

Traits	QTLs	Permutation threshold^a^	Env	Chr.	Nearest marker	Distance(cM)	LOD	additive^b^	R^2^ ^c^	source
LC	*q*LC-7-1	2.94	1	7	NAU1043	1	10.34	3.075	42.83	Zong128
			2	7	NAU1043	1	10.34	3.36	49.82	Zong128
			3	7	NAU1043	1	9.62	2.976	43.16	Zong128
			4	7	NAU1043	1	9.63	2.78	37.88	Zong128
FC	*q*FC-7-1	3.11	1	7	NAU1043	2.01	9.47	3.235	50.19	Zong128
			2	7	NAU1043	1	9.31	3.484	59.85	Zong128
			3	7	NAU1043	2.01	9.69	3.54	49.91	Zong128
			4	7	NAU1043	2.01	8.58	2.915	40.05	Zong128
FL	*q*FL-1-1	2.6	1	1	NAU4073	4.75	2.68	-0.6292	5.83	KucheT94
			2	1	NAU4073	2.75	3.14	-0.519	3.75	KucheT94
	*q*FL-6-1	2.7	1	6	DPL681	11.29	6.64	1.137	5.48	Zong128
			2	6	DPL681	9.29	3.54	0.653	3.73	Zong128
			3	6	DPL681	11.29	5.72	0.8602	3.78	Zong128
			4	6	DPL681	11.29	4.5	0.7551	3.75	Zong128
	*q*FL-6-2	2.65	2	6	DPL681	2.01	3.85	0.5559	3.84	Zong128
	*q*FL-7-1	2.56	1	7	NAU1043	1	4.04	-0.6929	5.88	KucheT94
			2	7	NAU1043	1	4.68	-0.5999	3.82	KucheT94
			3	7	NAU1043	1	3.5	-0.5234	3.92	KucheT94
			4	7	NAU1043	1	3.57	-0.5277	3.85	KucheT94
FU	*q*FU-6-1	2.63	2	6	BNL1440(b)	2.17	2.62	0.3768	2.7	Zong128
			3	6	BNL1440(b)	2.17	2.93	0.3726	2.12	Zong128
	*q*FU-7-1	2.68	1	7	NAU1043	1	7.89	-0.7559	3.45	KucheT94
			2	7	NAU1043	1	5.83	-0.5746	2.75	KucheT94
			3	7	NAU1043	1	4	-0.4206	2.19	KucheT94
			4	7	NAU1043	1	4.51	-0.7137	5.43	KucheT94
FF	*q*FF-7-1	2.9	1	7	NAU1043	1	4.5	-0.2161	5.15	KucheT94
	*q*FF-24-1	3.02	2	24	NAU1322	2.01	2.94	0.12	2.48	Zong128
			3	24	NAU1322	2.01	3.5	0.191	4.38	Zong128
			4	24	NAU1322	2.05	4.3	0.1545	2.41	Zong128
FE	*q*FE-7-1	2.57	2	7	NAU1043	1	3.15	0.2048	6.72	Zong128
	*q*FE-9-1	2.96	2	9	NAU3888	12.14	7.89	-0.5085	6.05	KucheT94
			3	9	NAU3888	10.14	3.74	-0.3558	4.74	KucheT94
			4	9	NAU3888	10.14	8.14	-0.4769	4.83	KucheT94
	*q*FE-9-2	4.68	2	9	NAU3674	1.84	9.98	-0.5289	6.6	KucheT94
			3	9	NAU3674	3.84	5.11	-0.3441	5.07	KucheT94
			4	9	NAU3674	3.84	11.25	-0.516	4.98	KucheT94
	*q*FE-12-1	2.67	3	12	NAU2672	5	3.13	0.2	5.02	Zong128
	*q*FE-23-1	2.73	3	23	NAU3025	7.69	3.07	0.2877	4.81	Zong128
			4	23	NAU3025	5.69	4.99	0.3758	4.78	Zong128
FS	*q*FS-6-1	2.62	1	6	DPL681	11.29	3.62	0.95	7.95	Zong128
			2	6	DPL681	7.29	2.75	0.8225	8.69	Zong128
			3	6	DPL681	7.29	3.73	0.9792	8.2	Zong128
			4	6	DPL681	5.29	3.36	0.8289	7.47	Zong128
	*q*FS-6-2	2.57	1	6	DPL681	2.01	3.02	0.8034	8.19	Zong128
			2	6	DPL681	2.01	3.83	0.8644	8.62	Zong128
			3	6	DPL681	2.01	4.92	0.9646	8.3	Zong128
			4	6	DPL681	2.01	3.91	0.9266	7.31	Zong128
	*q*FS-7-1	2.77	1	7	NAU1043	1	7.17	-1.113	8.38	KucheT94
			2	7	NAU1043	1	6.23	-1.0654	8.78	KucheT94
			3	7	NAU1043	1	5.66	-1.0021	8.49	KucheT94
			4	7	NAU1043	1	4.41	-0.8298	7.42	KucheT94
	*q*FS-24-1	2.81	2	24	NAU1322	2.01	3.07	0.7295	8.73	Zong128
			3	24	NAU1322	2.01	2.76	0.6733	8.4	Zong128
			4	24	NAU1322	2.01	3.12	0.6792	7.38	Zong128

Note: LC: lint color, FC: fuzz color, FL: fiber length, FU: fiber length uniformity, FS: fiber strength, FE: fiber elongation, FF: fiber fineness (micronaire value).

^a^ The means of significant LOD threshold for each trait over four or two environments at P = 0.05 determined by 1,000 permutation test

^b^ Additive effect. A positive value means the female parent (Zong128) having a positive effect on the trait. A negative value means the male parent (KucheT94-4) having a positive effect on the trait.

^c^ Phenotypic variation explained by a single QTL. Environment = different locations and years in which the RILs were planted: 1, 2009 in Anyang; 2, 2010 in Anyang; 3, 2010 in Huangmei; 4, 2010 in Shihezi.

#### Fiber quality

Based on composite interval mapping, 17 QTLs for five fiber quality traits were detected (Table 1, Fig 2). These QTLs were mapped on seven chromosomes, and six QTLs were detected in all four environments.

For fiber length, four QTLs (Table 1, Fig 2) were identified and mapped on three chromosomes. The *q*FL-6-2 and *q*FL-1-1 were identified in one or two environment explainining 3.84% and 3.75–5.84% of the PV, respectively. While *q*FL-6-1 and *q*FL-7-1 were identified in all four environments, explained 3.73–5.48% and 3.75–5.84% of the PV, respectively. However, *q*FL-7-1 had the same location as the lint color QTL *q*LC-7-1, but showed negative additive effect across four environments. Longer fiber length was conferred by an allele on chromosome 1 and chromosome 7 from the long-fibered parent KucheT94-4, while shorter length was associated with alleles on chromosome 6 from the short-fibered parent Zong128.

Two QTLs for fiber length uniformity (Table 1, Fig 2) were identified and mapped on chromosome 6 and chromosome 7, showing minor genetic effects. The *q*FU-6-1 was identified in two environments with a positive additive effect, and explained 2.7% (LOD 2.62) and 2.12% (LOD 2.93) of the PV, respectively. The *q*FU-7-1 showed a negative additive effect with 3.45%, 2.75%, 2.19% and 5.43% of the PV in four environments, respectively. And it was identified at the same location as *q*LC-7-1, separating from the marker NAU1043 by 1.0 cM.

Two QTLs for fiber fineness (Micronaire value) (Table 1, Fig 2) were identified and mapped on chromosome 7 and chromosome 24. *q*FF-7-1 identified in one environment only, and explained 5.15% of the PV with a LOD score of 4.5. The Zong128 allele conferred an additive effect of -0.2161 at this QTL. While, *q*FF-24-1 had a positive additive effect accounting for 2.48% (LOD 2.94), 4.38% (LOD 3.5), and 2.41% (LOD 4.3) of the PV in three environments.

Five QTLs for fiber elongation were detected and mapped on chromosomes 7, 9, 12 and 23, accounting for 4.74 to 6.72% of the PV (Table 1, Fig 2). But none of the QTLs was identified in all four environments. *q*FE-9-1 and *q*FE-9-2 were identified in three environments, both of which had a negative additive effect. While the other three QTLs had a positive additive effect. Co-located with the lint color QTL *q*LC-7-1, *q*FE-7-1 conferred a positive additive effect of 0.2048, and identified in one environment only explaining 6.72% (LOD 3.15) of the PV.

For fiber strength, four QTLs were detected on chromosomes 6, 7 and 24, accounting for 7.31 to 8.78% of the PV (Table 1, Fig 2). The *q*FS-24-1 was identified in three environments, while *q*FS-6-1, *q*FS-6-2, and *q*FS-7-1 were identified in four environments. Only qFS-7-1 conferred a negative additive effect by -1.1130, -1.065, -1.0021 and -0.8298, respectively.

### Comparison of the QTLs for fiber color and quality traits between the different F_2_ and RIL populations

There were 13 QTLs for fiber color and quality traits detected in the F_2_ population of Zong128 × KucheT94-4 from which the RIL population has also been developed (Table 2 and Fig 3). Seventeen QTLs for fiber color and quality traits were detected in the F_2_ population of Zong128 × Liao96-23-30 (Table 3 and Fig 4). Four QTLs, *q*LC-7-1, *q*FC-7-1, *q*FL-7-1, and *q*FS-7-1, were all identified in the two F_2_ populations and the RILs. The *q*FU-7-1 was identified both in F_2_ and RIL populations of Zong128 × KucheT94-4 with negative addition effect. The QTL *q*FE-7-1 were detected in the F_2_ population of Zong128 × Liao96-23-30 with a positive additive effect, but detected in the RIL population of Zong128 × KucheT94-4 with a negative additive effect. The QTL *q*LC-7-1, *q*FC-7-1, *q*FL-7-1, *q*FS-7-1, *q*FU-7-1, and *q*FE-7-1 had the same location flanking by NAU1043 and NAU3654, and separated with NAU1043 by a distance of 1.0 cM. Thus, it may be a multiple-effect locus for fiber quality and fiber color but the alleles of this locus showed positive hereditary effects in fiber color, but negative hereditary effects in fiber quality traits. These alleles of multiple-effect locus carried in the brown fiber line, Zong128 had a strong synergism effect for the color of fiber lint and fuzz (*q*LC-7-1 and *q*FC-7-1), but decreased the fiber length (*q*FL-7-1), strength (*q*FS-7-1) and uniformity (*q*FU-7-1) in the RIL and F_2_ population of the across Zong128 × KucheT94-4 and in F_2_ population of Zong128 × Liao96-23-30. However, the alleles of *q*LC-7-1, *q*FC-7-1, *q*FL-7-1, *q*FS-7-1 and *q*FU-7-1 originated from another parent KucheT94-4 with white fiber had the reverse effects in fiber color and fiber quality compared with those from the brown fiber parent Zong128. The alleles of *q*FL-6-2, *q*FS-6-2 and *q*FU-6-1 between DPL681 and BNL1440(b) originated from the brown fiber line Zong128 can increase the fiber length (*q*FL-6-2) and strength (*q*FS-6-2) in the RIL population. This indicated that one SSR locus may affect the multiple traits of the fiber (lint and fuzz) color and their qualities, and this may resulted in the linked inheritance of different traits despite of the positive or negative effects of alleles.

**Table 2:** QTLs detected for fiber color and quality traits in F_2_ population of Zong128 × KuchuT94-4.

Traits	QTLs	Chr.	Nearest marker	Distance (cM)	LOD	Additive^a^	Dominant^b^	R^2^ ^c^	Additiive source
LC	*q*LC-5-1	5	NAU3273	4.01	7.96	-0.246	-0.735	0.44	KucheT94-4
	*q*LC-7-1	7	NAU1043	1	27.23	4.86	-0.312	27.37	Zong128
	*q*LC-14-1	14	NAU4022	2.23	7.78	0.2127	-0.365	0.39	Zong128
	*q*LC-24-1	24	NAU3255	8.01	5.65	-1.572	-0.878	2.43	KucheT94-4
FC	*q*FC-5-1	5	NAU3273	4.01	9.79	-0.207	-0.81	0.31	KucheT94-4
	*q*FC-7-1	7	NAU1043	1	28.76	6.45	-0.298	47.46	Zong128
	*q*FC-14-1	14	NAU4022	4.01	9.72	0.362	-0.602	0.78	Zong128
	*q*FC-24-1	24	NAU3255	6.01	5.52	-1.426	-1.119	1.97	KucheT94-4
FL	*q*FL-5-1	5	NAU3273	1	4.17	0.268	0.189	2.28	Zong128
	*q*FL-7-1	7	NAU1043	1	6.09	-1.3211	0.3046	11.87	KucheT94-4
FU	*q*FU-7-1	7	NAU1043	1	7.19	-1.2088	0.3782	14.71	KucheT94-4
FE	*q*FF-6-1	6	BNL1440(b)	7.85	3.42	0.2994	-0.4768	4.92	Zong128
FS	*q*FS-7-1	7	NAU1043	1	3.44	-1.154	0.2194	6.87	KucheT94-4

Note: LC: lint color, FC: fuzz color, FL: fiber length, FU: fiber length uniformity, FS: fiber strength, FE: fiber elongation, FF: fiber fineness (micronaire value).

^a^ Additive effect. A positive value means the female parent (Zong128) having a positive effect on the trait. A negative value means male parent (KucheT94-4) having a positive effect on the trait.

^b^ Dominant effect

^c^ Phenotypic variation explained by a single QTL.

**Table 3:** QTLs detected for fiber color and quality traits in F_2_ population of Zong128 × Liao96-23-30.

Traits	QTLs	Chr.	Nearest marker	Distance (cM)	LOD	Additive^a^	Dominant^b^	R^2^ ^c^	additve source
LC	*q*LC-7-1	7	NAU1043	1	7.03	3.07	-1.25	16.01	Zong128
	*q*LC-21-1	21	BNL1551	6.01	12.08	0.453	-0.32	2.04	Zong128
	*q*LC-21-2	21	TMB1075	0.61	5.14	-0.34	0.1	0.91	Liao96-23-30
	*q*LC-21-3	21	NAU4004	2.81	3.61	-0.22	0.1	0.45	Liao96-23-30
FC	*q*FC-7-1	7	NAU1043	1	7.67	3.29	-1.4	17.34	Zong128
	*q*FC-21-1	21	BNL1551	6.01	11.93	0.464	-0.33	2.39	Zong128
	*q*FC-21-2	21	TMB1075	0.61	5.19	-0.36	0.105	0.94	Liao96-23-30
FL	*q*FL-1/15-1	1\15	NAU2083	0.3	2.89	-1.246	1.4737	6.18	Liao96-23-30
	*q*FL-5/19-1	5\19	TMB1750	3.97	5.65	-0.913	0.8807	11.7	Liao96-23-30
	*q*FL-5/19-2	5\19	HAU432	1	7.22	-0.999	0.8919	14.04	Liao96-23-30
	*q*FL-5/19-3	5\19	MUCS127	4.01	6.89	-1.049	1.176	14.14	Liao96-23-30
	*q*FL-7-1	7	NAU1043	1	3.07	-0.833	0.6603	6.9	Liao96-23-30
FU	*q*FU-14-1	14	NAU6280	0.06	3.07	1.1003	-0.3724	12.14	Zong128
FF	*q*FF-14-1	14	NAU4022	3.69	2.5	-0.153	0.1213	2.28	Liao96-23-30
FE	*q*FE-7-1	7	NAU1043	1	3.22	-0.122	0.0746	7.86	Liao96-23-30
	*q*FE-14-1	14	NAU4022	2.01	2.5	0.028	-0.054	0.02	Zong128
FS	*q*FS-7-1	7	NAU1043	1	2.76	-1.076	0.7144	6.9	Liao96-23-30

Note: LC: lint color, FC: fuzz color, FL: fiber length, FU: fiber length uniformity, FS: fiber strength, FE: fiber elongation, FF: fiber fineness (micronaire value).

^a^ Additive effect. A positive value means the female parent (Zong128) having a positive effect on the trait. A negative value means the male parent (Liao96-23-30) having a positive effect on the trait.

^b^ Dominant effect

^c^ Phenotypic variation explained by a single QTL.

The major QTL for fiber color also appeared in the two F_2_ populations, but with a lower genetic effect in PV than that in the RIL population. The PV of *q*LC-7-1 was two times lower in the F_2_ of Zong128 × KucheT94-4, which had a light brown fiber color in the F_1_, than that in the RILs of Zong128 × KucheT94-4, which had dark brown fiber color in the offspring. Similarly, the PV of *q*LC-7-1 was three times lower in the F_2_ of Zong128 × Liao96-23-30, which had a nearly light fiber color in the F_1_, than that in the RILs of Zong128 × KucheT94-4. Compared to the RIL population, several QTLs excepting for *q*LC-7-1 and *q*FC-7-1 for lint and fuzz color showed minor genetic effects in the two F_2_ populations of Zong128 × KucheT94-4 and Zong128 × Liao96-23-30. Moreover, these QTLs were different between the two F_2_ populations. Three different lint color QTLs (*q*LC-5-1, *q*LC-14-1 and *q*LC-24-1) and three fuzz color QTLs (*q*FC-5-1, *q*FC-14-1 and *q*FC-24-1) were detected on chromosomes 5, 14 and 24 explaining 0.31–2.43% of phenotypic variation in the F_2_ of Zong128 × KucheT94-4 (Table 2 and Fig 3). Other lint color QTL (*q*LC-21-1, *q*LC-21-2 and *q*LC-21-3) and fuzz color QTL (*q*FC-21-1 and *q*FC-21-2) were located on chromosome 21 explaining 0.45–2.39% of phenotypic variation in F_2_ of Zong128 × Liao96-23-30 (Table 3 and Fig 4). These results indicate that the fiber color is mainly controlled by the major QTLs located on A genome (chromosome 7), and some minor genetic QTLs located on D genome (14, 21 and 24). In addition, the two QTLs *q*LC-7-1 and *q*FC-7-1 had negative dominant effects in the two F_2_ populations respectively.

## Discussion

### Low polymorphism and distorted segregation

Low polymorphism rates of molecular makers were found in the previous research. It was reported that out of 4,106 surveyed SSR primers pairs, only 122 (2.97%) were polymorphic in RIL population [39]. Similar low polymorphism rates (2.26%) of the intra-specific crosses in upland cotton were observed in F_2_ populations screened with SSR [41]. Low-DNA marker polymorphism among varieties [42] was a major hindrance of genetic map construction affecting the genome coverage and marker density in intra-specific upland cotton. In this study, we used currently available SSR markers susceptible to be associated with fiber color. Meanwhile, this experiment was focused on understanding of fiber color variation in the F_1_ crossed from Zong128 with different white fiber line, and explaining why fiber color and quality traits were in negative correlation for cotton genetic improvement.

In addition to the low polymorphism, we found highly skewed deviation from Mendelian ratios or segregation distortion in the mapping populations. Segregation distortion is an ubiquitous phenomenon whose extent, origin and genetic effects vary significantly with species, population types, crosses and marker types in plants [43]. Segregation distortion is mainly caused by pollen tube competition, pollen killer genes, selective fertilization (gametophytic or zygotic selection), abortion and chromosome translocation [44–45] or genetic drift cytological attributes, or biological reason [30]. The segregation distortion can reach extreme values in inter-specific crosses, most likely due to divergence between species in cotton [17, 20, 46]. Similar observations have also been reported in intraspecific crosses by Li et al. [45] who found 77.24% of distorted markers in Asian cotton (*Gossypium arboretum*).

In this study, the mapped QTLs in intraspecific crosses displayed high frequency of distorted markers. The F_2_ population of Zong128 × KucheT94-4 displayed higher segregation distortion rate than that of its related recombinant inbred lines, which was consistent with previous reported [43, 47].

### The location of fiber color QTLs

Fiber color heredity has been scrutinized by numerous authors, and determined by one or more major genes [3, 6, 7, 24], or with some minor genes [7, 24]. But, a very few scholars pay attention to the inheritance of the color on lint and fuzz on simultaneous basis. Shi *et al*. [3] concluded that colored cotton fiber lint and fuzz were controlled by one pair of major genes incomplete in dominance on non-homologous chromosomes, respectively. Also, there exists genetic interaction between fiber lint and fuzz coloring genes. However, it was reported that the brown fiber lint and fuzz were separately controlled by a major pair of dominant genes and two pairs of minor genes [24]. In this study, we mapped QTLs for brown fiber and detected two major QTLs (*q*LC-7-1 and *q*FC-7-1) on chromosome 7, which were stably expressed in multiple environments and three populations with large phenotypic variances. Thus, these major QTLs (*q*LC-7-1 and *q*FC-7) could be suitable for MAS-breeding in brown cotton. The minor genes could not be detected in RIL population, which may have been concealed due to the pure lines genes. However, they were identified in the two F_2_ populations. The *q*LC-21-2, *q*LC-21-3 and *q*FC-21-2 were located on chromosome 21 in the F_2_ of Zong128×Liao96-23-30, while the *q*LC-5-1, *q*FC-5-1, *q*LC-24-1 and *q*FC-24-1 were found on chromosomes 5 and 24 in another F_2_ population of the Zong128×KucheT94-4.

The qLC-7-1 and qFC-7-1, major QTLs for fiber lint and fuzz color were located between flanking markers NAU1043 and NAU3654 on chromosome 7. In fact, the lint and fuzz color were always the same for one genotype, which suggests that the traits are linked. This indicates that these lint and fuzz color QTLs were detected on the same chromosome region in the A genome. Coincidentally, the brown fiber gene LC1 was previously located on chromosome 7. *GhTT2-3* was a TT2 homozygous gene related to the brown fiber trait, co-segregated with the brown fiber gene (LC1) on chromosome 7 in a RIL population of T586 × Yumian 1 [48]. In white fiber cotton, the QTL qFB-LG05-1 of Yellowness (FB) associated with NAU1043 locus was located on chromosome 7 [34].

Thus, it is clear that the major fiber color QTL originating from the colored fiber parent are mainly distributed on the A genome, and the minor fiber color QTLs from the white fiber parent are located on the D genome (chromosomes 14, 21 and 24). The genetic effects of the minor QTL from the D genome were different. The fiber color variation was determined by both small and large genetic effects in the PVs of the major and minor QTL.

### The parents with white fiber also affected the fiber color in their offspring

It is interesting that the fiber color varied from brown, light brown, to nearly white depending on the different white fiber parents in the F_1_ population with common brown parent, Zong128. The fiber color of F_1_ from Zong128 × KucheT94-4 was light brown, but that of F_1_ from Zong128 × Liao96-23-30 was nearly white. As the fiber color of the female parent Zong128 was brown, the difference of the F_1_ fiber color may result from the male parent. The phenotype of the male parent of KucheT94-4 and Liao96-23-30 was white, but the genotype was different. Through a complete diallel-cross analysis of the two brown fiber and three white fiber lines, we considered that the different fiber colors of F_1_ generation may derive the additive effects of the different lines [9].

Four QTLs were detected on chromosomes 5, 7, 14 and 24 in the F_2_ population of Zong128 × KucheT94-4. The QTLs of *q*LC-7-1, *q*FC-7-1, *q*LC-14-1 and *q*FC-14-1 originated from the white cotton (KucheT94-4) decreased the fiber color, but *q*LC-5-1, *q*FC-5-1, *q*LC-24-1 and *q*FC-24-1 from this white parent increased the fiber color at 0.44% (additive effect: -0.246), 2.43% (additive effect: -1.572), 0.31% (additive effect: -0.207) and 1.97% (additive effect: -0.1.426) of the PV. Moreover, in the F_2_ population of Zong128 × Liao96-23-30, the QTL *q*LC-7-1, *q*FC-7-1, *q*LC-21-1 and *q*FC-21-1 from the white cotton parent Liao96-23-30 also decreased the fiber color. The QTLs *q*LC-21-2, *q*FC-21-2 and *q*LC-21-3 improved the fiber color with a smaller phenotype variation at 0.91%, 0.94% and 0.45% respectively compared with those QTLs in the F_2_ population of Zong128 × KucheT94-4. This indicated that QTLs or genes related to fiber color are also present in the white cotton, and these genes show different additive effects depending on different cultivars or lines.

In addition, we found transgressive segregation for fiber color in the three populations developed from brown and white cotton (S1 Table). Thus, the white cotton possesses genes that regulate fiber color. These genes had an undetectable effect on white fiber phenotype in the white parent KucheT94-4 and Liao96-23-30. However, their presence resulted in dark brown color beyond that determined by the major genes (*q*LC-7-1and *q*FC-7-1) and indicated an epistatic interaction that strengthened the brown phenotype.

### Multiple-effects locus for fiber qualities and fiber color

QTLs for fiber qualities have been identified and assigned to chromosomes or sub-genomes in earlier reports on inter-specific and intra-specific populations. These QTLs may represent major QTLs for fiber quality traits and they could be used in MAS. In our research, five QTLs (*q*FL-7-1, *q*FS-7-1, *q*FU-7-1, *q*FF-7-1 and *q*FE-7-1) for fiber quality traits were identified at the same locus for fiber color QTLs (*q*LC-7-1, *q*FC-7-1) distant from the marker NAU1043 by1.0 cM. The fiber color QTLs (*q*LC-7-1, *q*FC-7-1) had very high phenotypic variation and mainly originated from the brown parent Zong128. However, the QTLs qFL-7-1, qFS-7-1, qFU-7-1 and qFF-7-1 for the fiber quality traits had negative effects on the brown fiber cotton (Zong128). This indicates that the allele for QTLs from the locus of NAU1043 on chromosome 7 has multiple effects on fiber color and fiber quality. Similarly, it was reported that the QTLs for yellowness qFB-LG05-1 and fiber strength qFS-LG05-1 associated with NAU1043 were also detected on chromosome 7 [35]. Five QTLs (FL2, FU2, FS2, FE1 and FF1) were detected on chromosome 7 in the F_2:3_ populations of T586 × Yumian 1 and closely linked with the genetic marker gene (*LC*_*1*_) of fiber color [20]. This also indicates that the locus of *LC*_*1*_ could affect cotton fiber quality. The multiple-effect locus explained why fiber strength and length were negatively correlated with cotton brown color.

### Analysis of qualitative trait loci and their QTL effect

Typically, qualitative and quantitative traits are analyzed by different genetic models for the phenotype and genotype study. A significant negative kurtosis for fiber lint color (LC) and fuzz color (FC) in four environments in RILs population showed that the two traits were not normally distributed (absolute value >1). It seemed that LC and FC were not following quantitative traits pattern. However, these two traits had a normal distribution in the Zong128 × KucheT94-4 and Zong128 × Liao96-23-30 F_2_ populations, which indicated that the LC and FC were not controlled by a single gene. Therefore, we have used the QTL method to analyze the two traits in different F_2_ population and their related RIL population to compare the fiber color and quality traits in one genetic module.

The genetic effects of fiber lint color and quality of brown cotton had been analyzed through the complete 5×5 diallel crossing. We found that fiber color had significant negative correlation with fiber yield and quality (lint percentage, fiber length, uniformity, elongation and strength) [9]. Moreover, the QTLs for the LC and FC had larger PV (40−50%) in the RIL population across multiple environments. On the other hand, the fiber length QTL and the fiber strength QTL explained 3.75−5.88% and 7.31−8.78% of the PV respectively. Therefore, there are obvious differences in the fiber color and fiber quality traits inheritance. We consider that fiber lint color and fuzz color were controlled by oligogenic inheritance while polygenic inheritance regulated fiber length and fiber strength.

We hypothesized that incomplete quantitative traits should have large genetic effects in PV (>30%) and the complete quantitative traits should have genetic effects in PV (<10%) attributed to polygenic effects. The traits with QTL that explained 10−30% of the PV should be considered as partial quantitative traits.

## Supporting Information

S1 Table Phenotypic analysis of fiber color and quality for the parents and three populations. Note: 1, 2008 in Anyang; 2, 2009 in Anyang; 3, 2009 in Huangmei; 4, 2009 in Shihezi; LC: lint color, FC: fuzz color, FL: fiber length, FU: fiber length uniformity, FS: fiber strength, FE: fiber elongation, FF: fiber fineness (micronaire value).
(XLSX)
